# Performance of a Community-based Health and Nutrition-education Intervention in the Management of Diarrhoea in a Slum of Delhi, India

**DOI:** 10.3329/jhpn.v28i6.6603

**Published:** 2010-12

**Authors:** Smriti Pahwa, Geeta Trilok Kumar, G.S. Toteja

**Affiliations:** ^1^ Institute of Home Economics, Delhi University, New Delhi 110 016, India; ^2^ Indian Council of Medical Research, New Delhi, India

**Keywords:** Community health, Diarrhoea, Interventions, Nutrition education, Oral rehydration solutions, Slums, India

## Abstract

Diarrhoeal infections are the fifth leading cause of death worldwide and continue to take a high toll on child health. Mushrooming of slums due to continuous urbanization has made diarrhoea one of the biggest public-health challenges in metropolitan cities in India. The objective of the study was to carry out a community-based health and nutrition-education intervention, focusing on several factors influencing child health with special emphasis on diarrhoea, in a slum of Delhi, India. Mothers (n=370) of children, aged >12–71 months, identified by a door-to-door survey from a large urban slum, were enrolled in the study in two groups, i.e. control and intervention. To ensure minimal group interaction, enrollment for the control and intervention groups was done purposively from two extreme ends of the slum cluster. Baseline assessment of knowledge, attitudes, and practices on diarrhoea-related issues, such as oral rehydration therapy (ORT), oral rehydration salt (ORS), and continuation of breastfeeding during diarrhoea, was carried out using a pretested questionnaire. Thereafter, mothers (n=195) from the intervention area were provided health and nutrition education through fortnightly contacts achieved by two approaches developed for the study—‘personal discussion sessions’ and ‘lane approach’. The mothers (n=175) from the control area were not contacted. After the intervention, there was a significant (p=0.000) improvement in acquaintance to the term ‘ORS’ (65–98%), along with its method of reconstitution from packets (13–69%); preparation of home-made sugar-salt solution (10–74%); role of both in the prevention of dehydration (30–74%) and importance of their daily preparation (74–96%); and continuation of breastfeeding during diarrhoea (47–90%) in the intervention area. Sensitivity about age-specific feeding of ORS also improved significantly (p=0.000) from 13% to 88%. The reported usage of ORS packets and sugar-salt solution improved significantly from 12% to 65% (p=0.000) and 12% to 75% (p=0.005) respectively. The results showed that health and nutrition-education intervention improved the knowledge and attitudes of mothers. The results indicate a need for intensive programmes, especially directed towards urban slums to further improve the usage of oral rehydration therapy.

## INTRODUCTION

Diarrhoea is a leading cause of mortality of children aged less than five years (under-five children) globally. More than 1.5 million under-five children still continue to die each year due to acute diarrhoea (1). The number of these deaths can be substantially reduced by simple remedies, such as rehydration with oral rehydration salt (ORS) and fluids available in the home, continued feeding during diarrhoeal episode, and breastfeeding. However, less than 40% of children with diarrhoea in developing countries receive the recommended treatment, a trend which has made very little progress in the last decade (2, 3). As per the National Family Health Survey (NFHS) in India, 48% of children suffering from diarrhoea received oral rehydration therapy (ORT) (4). Thus, diarrhoea is one of the big public-health challenges, particularly in the unhygienic environment of rapidly-growing urban slums. While, on one hand, effective addressing of the health issues of the urban poor may take some time, the urgency of attempting immediate simple interventions is being realized (5, 6). One such attempt was made by the authors of this paper.

A community-based health and nutrition-education intervention focusing on several factors influencing child health in urban slums was carried out in Delhi, India. Diarrhoea received special emphasis in the intervention because not only do the maximum number of child deaths due to diarrhoea occur in India but it is also a major child-health issue in urban slums of the country (7, 8). Although it is known that knowledge, attitudes and practices (KAPs) of mothers play a crucial role in terms of childhood diarrhoea, community-based interventions to improve these aspects are few. The present paper describes the intervention in terms of community-based approaches developed for educating mothers and their impact on some aspects of management of diarrhoea addressed in the intervention.

## MATERIALS AND METHODS

A community-based health and nutrition-education intervention study was conducted in an urban slum in Delhi, India. It involved the creation of a model for imparting health and nutrition education to mothers on various issues critical to the health of their children. Issues, such as infant-feeding practices, counselling on general diet for better nutritional status with special reference to vitamin A deficiency (VAD) (9), personal hygiene, and diarrhoea, were addressed. The present paper describes the intervention in terms of the approaches developed and their impact on diarrhoea-related aspects that were, inter alia, covered during the study.

### Locale

The study was conducted in a slum cluster located at Kirti Nagar area in West Delhi. The cluster is densely populated with approximately 7,000 *jhuggis* (small dwelling units, mostly single-room structures, made up of mud or concrete or a mixture of both, each of them not exceeding more than 15 square yards in area). The cluster is further divided into various subclusters, mostly referred to as camps. The constitution of these camps is similar in terms of demographic profile of the resident population. Inhabitants of the cluster are migrants from the eastern part of the country and from the neighbouring states. They are predominantly Hindus (96.2%) and live in nuclear families (76.8%) as opposed to joint families (23.2%). As against 41% illiteracy in the study population, 79.9% of women who formed the target sample for the study were illiterate; the majority (69.7%) of them were aged less than 29 years.

**Fig. 1.**
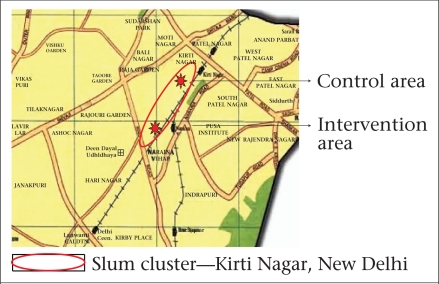
Location of slum cluster in Delhi, along with control and intervention areas within the cluster

Two areas—one treated as control (hereinafter referred to as control area) and the other used for intervention (referred to as intervention area)—were purposively selected from the two extreme ends of the slum (Fig. 1) to ensure minimal interaction between the subjects belonging to the control and intervention groups. These two slum subclusters—Jawahar camp (intervention area) and Harijan camp with Chuna Bhatti (control area, although a part of the same slum) were separated by a distance of approximately 2–3 km.

*The map is not to scale

### Target group

Target group for the study comprised mothers of children aged >12–71 months.

*Inclusion and exclusion criteria:* Mothers, who were permanent residents of the area, not working outside their homes and were willing to participate, were enrolled for the study while those likely to shift from the cluster either to some other places or back to their native villages or those working outside their houses were excluded.

*Sampling:* The study intended to offer health and nutrition education on some most critical issues affecting the health of children in Indian slums. This research formed the part of a study which involved looking at the effect of educational intervention on vitamin A status of children aged >12–71 months, the sampling for which was based on mean serum retinol. The sample-size of 300 (150 each for control and intervention groups) was calculated, assuming a 1.8% change in the mean retinol level at 95% confidence interval and 90% power. Assuming a drop-out of approximately 20%, 370 mother-child pairs were enrolled (175 from the control area and 195 from the intervention area).

*Enrollment:* With the help of health functionaries of the area, sketch of the subclusters was made. Sampling units in each subcluster were identified as per standard technique of identifying the point of random start by preparing a sketch of the study area (10). After the start of the survey as soon as the households having children aged >12–71 months were identified, the purpose of the study was explained to mothers and other family members. Once they agreed, written consent in the local language was given, the mother was enrolled for the study, and the *jhuggi* was marked with an identification sign. This enrollment procedure was followed until the required sample-size was met. The identification survey and enrollment had to be done simultaneously due to paucity of funds.

### Study design

The study was carried out in three phases: baseline survey, intervention, and repeat survey. During the baseline survey, which was carried out over a three-month period, a questionnaire, suitably designed and pretested, was used for collecting information on knowledge, attitudes, and practices of mothers regarding the key health and nutrition issues. With respect to diarrhoea, the questionnaire explored the acquaintance of mothers with the term ORS or *Jeevan Rakshak Ghol*—its local equivalent. The level of knowledge was tested on the issues, such as importance and need of ORT during diarrhoea; correct method of preparation, usage, and feeding of ORS available as packets and home-made sugar-salt solution; and continuation of breastfeeding during diarrhoea. Attitudes, of mothers, in terms of whether they felt that the amount of ORS fed to the child was also important, was assessed using a three-point Likert's scale (11). This scale had three categories of responses: ‘agree’, ‘disagree’, or ‘uncertain’. Every mother was presented with some statements, and their responses in terms of whether they agreed or disagreed or were uncertain about the statement were recorded as per the scale. The questionnaire also included questions on the most common practices as reported by mothers, in case their children suffered from diarrhoea.

After the completion of the baseline survey, key diarrhoea-related issues that needed intervention were identified, and the intervention was planned accordingly. Detailed lesson plans were developed, specifying the objectives to be achieved, messages to be given, aids to be used, and activities to be undertaken for each topic covered under intervention. For diarrhoea, the objectives of education were to sensitize the mothers on the necessity of oral rehydration; make them understand the importance of continuing breastfeeding during diarrhoea; empower them with the knowledge of the importance, preparation, and use of ORS from the packets available and at home in the form of sugar-salt solution, and make them aware of the need of daily preparation of ORS.

Two approaches—‘personal discussion sessions’ and ‘lane approach’—were developed to impart education. In the first approach, the intervention group was divided into subgroups consisting each of 15–20 mothers. Personal discussions were held with each subgroup in a community centre located in the slum area. Each personal discussion session lasted for 30–45 minutes.

In the lane approach, *jhuggis* of mothers of each subgroup were visited lane-wise at an interval of about 15 days after the personal discussion session at the community centre. In this approach, 6–7 mothers residing in one lane were assembled in a *jhuggi,* and the main messages given in the discussion session were revised in a 15–20-minute interaction. As lane-visits were scheduled at an interval of 15 days from the discussion session in the community centre, almost every mother in the intervention group was contacted fortnightly during the intervention phase. Each group was contacted twice a month. One contact was made during the personal discussion sessions, and another contact was made by lane-visits.

The intervention phase lasted for nine months. During these nine months, each group comprising 15–20 mothers was contacted 18 times. Five of these contacts focused exclusively on diarrhoea. In rest of the contacts, key messages about diarrhoea were briefly reiterated.

### Methods for imparting health and nutrition education

Health and nutrition education was imparted using audiovisual media and demonstration and carrying out focus-group discussions (12). In addition to our aids, available educational material, in the form of posters, flips-charts, and flash-cards, developed by national and international agencies, was used for educating the community. The preparation of ORS was demonstrated, and the quantity required as per age of the child was explained. The usage of a simple home-made sugar-salt solution was emphasized, and its preparation was demonstrated by involving the peripheral health worker*—Basti Sevika*—in the community. A bottle with a small hole at the bottom filled with water was used for explaining the concept of dehydration in diarrhoea (13).

Handouts were prepared summarizing the key messages given during the intervention. As the target group had poor literacy rates, handouts were designed to deliver the information mostly through pictorial representation. These handouts were shown to the mothers during the discussion sessions and put up in the dwelling unit of each participating mother. The handouts were expected to reinforce all the concepts that were discussed during various contact points.

Peer learning was encouraged during the discussion sessions. Mothers were encouraged to explain the concepts to their group in presence of the investigator. In some personal discussion sessions, an active participant mother was asked to explain the concepts taught to the group. This mother was asked to explain the handouts to the group. Communicating with a fellow mother helped the remaining mothers in the group to express themselves and share their views without hesitation in their own local dialect. Moreover, this approach helped encourage more participation and hold the mother's interest in the discussion sessions.

A repeat survey was conducted after the intervention in the same three months in which the baseline survey was carried out the previous year.

### Analysis of data

Data were coded, entered in MS access, cleaned, and analyzed using the SPSS software (version 15.0) and the Microstat software (Microstat developed by Ecosoft. inc, 1984) for carrying out test of proportions. KAPs were reported in percentages. Data during the pre- and post-intervention phases were compared to assess the impact of health and nutrition education imparted.

## RESULTS

At baseline, 370 mothers of children, aged >12–71 months, were enrolled in the study (175 from the control area and 195 from the intervention area). After the intervention, 70 (19%) mothers were lost to follow-up, and 300 mothers (150 each from the control and intervention clusters) were interviewed during the repeat survey.

Table 1 summarizes information on awareness of mothers about ORS. At baseline, 71% of mothers from the control and intervention areas had heard about ORS whereas, at the time of the repeat survey, awareness of ORS among mothers from the intervention area increased significantly (p=0.000) from 65% to 98%. About 9% of mothers were aware of the correct method of reconstitution of ORS from the packets locally available, and 8% knew the correct method of preparation of the alternate sugar-salt solution at home for rehydration at baseline. These figures increased significantly (p=0.000) in the intervention area (ORS packets: 13–69%; sugar-salt solution: 10–74%) but not in the control area (ORS packets: 6–9%; sugar-salt solution: 7–9%) after the intervention.

**Table 1.** Awareness about ORS among slum mothers of Delhi

Control area				Intervention area				Baseline survey (1+2)	
Baseline survey (1)		Repeat survey		Baseline survey (2)		Repeat survey			
No.	%	No.	%	No.	%	No.	%	No.	%
Heard the term ‘ORS’									
n=175		n=150		n=195		n=150		n=370	
136	77.7	112	74.7	127	65.1	147	98.0	263	71.1
Aware that ORT prevents dehydration									
n=136*		n=112*		n=127*		n=147*		n=263*	
40	29.4	32	28.6	38	29.9	108	73.5	78	29.7
Knowing correct method for reconstitution of ORS from the packets locally available									
n=136*		n=112*		n=127*		n=147*		n=263*	
8	5.9	10	8.9	16	12.6	101	68.7	24	9.1
Knowing correct preparation of sugar-salt solution at home									
n=136*		n=112*		n=127*		n=147*		n=263*	
10	7.4	10	8.9	13	10.2	108	73.5	23	8.7
Aware that ORS has to be prepared fresh everyday									
n=136*		n=112*		n=127*		n=147*		n=263*	
106	77.9	89	79.5	94	74.0	141	95.9	200	76.0

*Number of mothers who were aware of ORS;

ORS=Oral rehydration salt

At baseline, 30% of mothers were aware that ORS was given to prevent dehydration while 51% thought that ORS would treat diarrhoea and, hence, did not take their children to doctor. In the intervention area, the education capsule was successful in significantly (p=0.000) increasing the awareness of the role of ORS in the prevention of dehydration (29–74%) (Table 1).

At baseline, 76% of mothers knew that ORS should be prepared fresh daily. In the post-intervention period, there was a further significant increase (from 74% to 96%, p=0.000) in the proportion of mothers who were aware of this in the intervention area (Table 1).

**Fig. 2.**
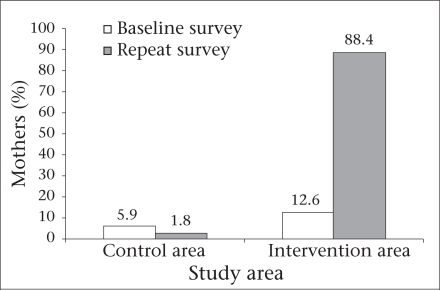
Mothers (%) who agreed that ORS has to be fed in specificamount as per child's age

Age-specific feeding of ORS was not considered relevant by most (91%) slum mothers at baseline. Intervention brought sensitization about the importance of feeding ORS in correct amounts. During the repeat survey, mothers in the intervention area, who felt the importance of feeding ORS in age-specific doses, rose significantly (from 13% to 88%; p=0.000). In the control area, there was a slight decline in the percentage of such mothers (Fig. 2).

ORS=Oral rehydration salt

The reported usage of commercially-available ORS packets and home-made sugar-salt solution among the study mothers at baseline was around 15% (ORS: 14.6%; sugar-salt solution: 15.4%). In the post-intervention period, the reported usage of ORS and sugar-salt solution increased significantly (p=0.001 and p=0.005 respectively) in the intervention area while no significant change was observed in the control area (Table 2).

**Table 2.** Diarrhoea-management practices in a Delhi slum

Practice	Control area				Intervention area				Baseline survey (n=370) (1+2)	
	Baseline survey (1) (n=175)		Repeat survey (n=150		Baseline survey (2) (n=195)		Repeat survey n=150)			
	No.	%	No.	%	No.	%	No.	%	No.	%
Give ORS	31	17.7	29	19.3	23	11.8	97	64.7	54	14.6
Give sugar-salt solution	33	18.9	29	19.3	24	12.3	112	74.7	57	15.4
Give fluids available at home	1	0.6	0	0.0	3	1.5	3	2.0	4	1.1
Go to a doctor	147	84.0	132	88.0	157	80.5	39	26.0	304	82.2
Nothing special	1	0.6	2	1.3	2	1.0	0	0.0	3	0.8
Any other	2	1.1	0	0.0	3	1.5	0	0.0	5	1.4
No response	0	0.0	0	0.0	0	0.0	0	0.0	0	0.0

ORS=Oral rehydration salt

**Fig. 3.**
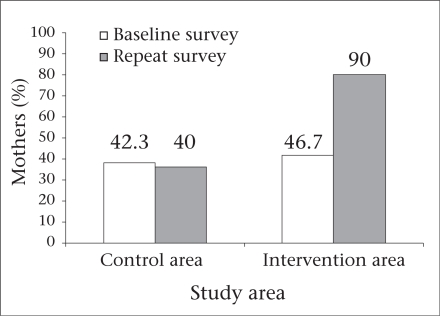
Mothers (%) of preschoolers in Delhi slum who were aware that child should be breastfed during diarrhoea

Awareness that breastfeeding should be continued during diarrhoea was only 47% at baseline in the slum cluster. After the intervention, 90% of mothers (p=0.000) in the intervention area said that breastfeeding should be continued during diarrhoea. In the control area, the proportion of such mothers remained almost similar (baseline: 42%; repeat survey: 40%) during the two surveys (Fig. 3).

## DISCUSSION

A community-based health and nutrition-education intervention was carried out in a slum cluster in New Delhi, with diarrhoea as one of the main focal points of the intervention. The study revealed that, in the post-intervention period, there was a significant (p=0.000) improvement in the acquaintance of mothers with the term ORS (65% vs 98%); correct method of reconstitution of ORS locally available (13% vs 69%) and preparation of sugar-salt solution (10 vs 74%); knowledge about the dehydration-preventing role of ORS (29.9 vs 73.5%); daily fresh preparation of ORS (74 vs 96%); and the importance of continuing breastfeeding during diarrhoea (47 vs 90%) in the intervention area. Sensitization about age-specific feeding of ORS also improved (from 13% to 88%; p=0.000). The reported usage of ORS packets and home-made sugar-salt solution improved significantly from 12% to 65% (p=0.000) and 12% to 75% (p=0.005) respectively.

Although the demonstrated improvement seems encouraging, it still has reservations given the limitations of the present study. One of the limitations of this study was its scale and purview with reference to diarrhoea, i.e. only a few very focused messages specific to childhood diarrhoea were disseminated. For assessing the impact, the study depended on the responses of mothers. Despite these limitations, the study highlights the importance of constant communication with the community and simple messages for health and nutrition education. The present study targeted primarily the awareness of mothers without making any attempt to address the issue of availability of ORS (if any) in the area or any other social, political, economic, infrastructural or operational issues that could affect childcare during diarrhoea. The simple input of education and community outreach seems to be beneficial in improving knowledge and hints the ushering in positive attitudes. There seems to be a possibility of a positive change in practices in terms of the increased reported usage of ORS and sugar-salt solution.

India accounts for more than half a million deaths due to diarrhoeal disease among under-five children (7). Diarrhoea is a major health problem among children in urban slums of the country (14). Poor environmental hygiene, coupled with low literacy level and poor awareness of residents, adversely affects the management of diarrhoea in slum areas and contributes to the burden of the disease (15). The Government of India views diarrhoea as a serious condition and has tried to address the issue since 1978 with its Diarrhoeal Diseases Control Programme. This was transformed into the National Oral Rehydration Therapy (ORT) Programme in 1985–1986 when it focused on strengthening the case management of diarrhoea in under-five children and improving maternal knowledge relating to the use of fluids available at home, use of ORS, and continued feeding. Since 1992–1993, the programme has become part of the Child Survival and Safe Motherhood (CSSM) Programme. Education of mothers and sensitization of the community about the causes, symptoms, and treatment of diarrhoeal disease have been emphasized by the Government of India. However, there is still a need to strengthen the health and nutrition-education initiatives (16).

The NFHS-3 revealed that, despite 63% of Indian mothers being aware of ORS, these packets are used only in 27% of cases of childhood diarrhoea. The survey revealed that, in slum areas of Delhi too, as high as 94% of women knew about ORS but their usage was just 39% (4). These data reveal a wide gap between the knowledge of ORS and its usage. Baseline data in the present study also highlight this disparity. This indicates that superficial knowledge of ORS is not enough. Concentrated efforts are required to highlight the importance of ORT in tackling dehydration during diarrhoea in children as they are more quickly dehydrated than adults and are, therefore, more likely to die of diarrhoea (17, 18). Replacing lost fluids and salts is a cheap and effective method of rehydration and can reduce a large proportion of deaths due to diarrhoea. Thus, the health and nutrition-education efforts need to go a step further so that they make caretakers realize the importance of ORT in diarrhoea (19). The correct knowledge of the preparation of ORS from the packets available or at home should be imparted to build the confidence of caretakers so that ORT is given to a child when suffering from diarrhoea. Proper feeding of ORS is also important. The present intervention imparted education on all these aspects. In the community, such educational interventions can be taken up in close collaboration with private ORS manufacturing pharmaceutical companies or local non-governmental organizations. The role of private partners has been highlighted in the latest seven-point plan for the comprehensive control of diarrhoea (1).

It was observed in the present study that, during the repeat survey, the level of knowledge among mothers in the control area dropped. This change could be attributed to the drop-out that occurred in the study sample with more knowledgeable mothers moving away from the slum or due to the lack of reinforcement of any knowledge that mothers in the control area possessed at the time of the base-line survey. Thus, it is felt that, although independent concentrated health and nutrition-education efforts are required, a quick reinforcement of these key issues during any contact with the community would help. In the model used in the present study, the contact through the lane approach was relatively brief but was instrumental in reinforcing the key messages given earlier. Similarly, during contacts of peripheral health workers with the community for any purpose, the community would definitely benefit if the health worker possesses a checklist for some key messages that are quickly reiterated. Further, rather than just maintaining a plain record of field-visits, it will be better to provide them with a structured checklist containing such key messages to make their field-visits more productive.

The present study was, thus, beneficial in improving the knowledge and attitudes of mothers. There seemed to be an improvement in the related practices as well, although extensive and extended studies which capture more concrete evidence of change are needed. Although the study was conducted in an urban slum, its essence in terms of the content of education and the approach can be of general use.
